# Molecular Evidence of Oysters as Vehicle of Norovirus GII.P17-GII.17

**DOI:** 10.3201/eid2211.161171

**Published:** 2016-11

**Authors:** Lasse Dam Rasmussen, Anna Charlotte Schultz, Katrine Uhrbrand, Tenna Jensen, Thea Kølsen Fischer

**Affiliations:** Statens Serum Institute, Copenhagen, Denmark (L.D. Rasmussen, T.K. Fischer);; Technical University of Denmark, Kongens Lyngby, Denmark (A.C. Schultz, K. Uhrbrand);; Danish Veterinary and Food Administration, Glostrup, Denmark (T. Jensen);; University of Southern Denmark, Odense, Denmark (T.K. Fischer)

**Keywords:** viruses, norovirus, oysters, gastroenteritis, Denmark, quasispecies, shellfish, food safety, viruses, enteric infections

**To the Editor:** Norovirus is the world’s leading cause of nonbacterial acute gastroenteritis (*1*). Since their emergence, GII.P17-GII.17 noroviruses have replaced the GII.4 Sydney 2012 variant as the dominating norovirus genotype in parts of Asia (*2*), although they have been detected only sporadically, in a limited number, on other continents (*3*).

The major reservoir(s) of GII.17 that contribute to transmission are unknown, but it has been suggested that oysters and other bivalve shellfish are common vehicles for transmission of the emerging GII.17 viruses (*2*). In this study, we demonstrate the link between oysters and human disease by presenting molecular evidence of norovirus GII.P17-GII.17 in Denmark causing acute gastroenteritis, characterized by the sudden onset of vomiting with or without diarrhea after consumption of oysters. We further document molecular evidence providing linkage between norovirus detected in fecal samples from patients and food samples from imported oysters.

During January 23–February 4, 2016, acute gastroenteritis developed in 58 of 67 persons who consumed oysters served on 18 separate occasions at 8 different restaurants and a private party, with onset of symptoms within 24–40 hours after the patients ate oysters. All oysters originated from 2 distinct oyster lots provided by 1 wholesaler in France and distributed by 1 wholesaler in Denmark. Oysters from both lots were harvested off the coast of La Rochelle, France.

In Denmark, submitting fecal samples in connection with foodborne outbreaks is voluntary. A total of 5 samples from 3 cases representing 2 different parties were submitted to the National Virus Surveillance Laboratory at Statens Serum Institut (Copenhagen, Denmark) for norovirus analysis. In addition, 4 samples of oysters from the same producer in France were sent to the National Food Institute, Technical University of Denmark (Kongens Lyngby, Denmark), for norovirus analysis. Two samples, an opened (sample A) box and an unopened (sample B) box collected at one of the restaurants involved in the outbreak, contained oysters from 1 batch (I), and another 2 samples (C and D) contained oysters from a separate batch (II) collected at the wholesale level.

Norovirus in fecal samples was detected and polymerase (open reading frame [ORF]1) and capsid (ORF2) gene regions were amplified as described elsewhere (*4*). Sequencing was performed by using an ABI 3500 genetic analyzer (Thermo Fisher Scientific, Nærum, Denmark). A fragment of 1,111 nt spanning the ORF1–ORF2 junction was amplified and sequenced by using forward ORF1 primer JV12 and reverse ORF2 primer G2SKR (*4*). Genotyping was performed by using the Web-based norovirus typing tool NoroNet (http://www.rivm.nl/mpf/norovirus/typingtool) (*5*).

We found that all fecal samples contained norovirus belonging to genogroup II (GII). Sequencing of the polymerase region was successful in 4 of the 5 samples; all 5 samples were sequenced in the capsid region. Typing of the sequences indicated GII.P17 and GII.17, respectively. Furthermore, PCR and sequencing of the long fragment covering the ORF1–ORF2 junction were successful in 2 samples, both genotyping as GII.P17-GII.17. Sequences had high homology (99.91%) to several Asian strains, such as Hu/GII.P17_GII.17/KR/2014/CAU-265 (*6*).

Oysters were analyzed for norovirus (*7*), and the capsid region of the detected strains was sequenced (*8*). Samples A, B, and C contained norovirus GI, whereas all 4 samples (A–D) contained norovirus GII. GI.2 was identified in 1 sample (B), and GII.17 was identified in all samples (A–D). The GII.17 sequences obtained from samples A–C showed 100% homology, whereas the sequence identified in sample D varied at 3 positions.

Comparisons of the capsid sequences obtained from 4 human samples and oyster samples A–C showed 100% homology. The last of the human sequences differed at 2 positions owing to mixed bases. The chromatogram showed equal intensities at both positions C and T (Figure). This finding indicates that quasispecies might be present. These positions in the human sequence coincided perfectly with 2 of the 3 positions found to vary in the sequence obtained from oyster sample D. All 3 base variations were a replacement of a C with a T (Figure), which further supports the presence of quasispecies. However, in this setup, it was not possible to prove the origin of these closely related species. Whole-genome sequencing using next-generation sequencing would be a way to prove the simultaneous presence of all quasispecies in relevant samples.

**Figure:**
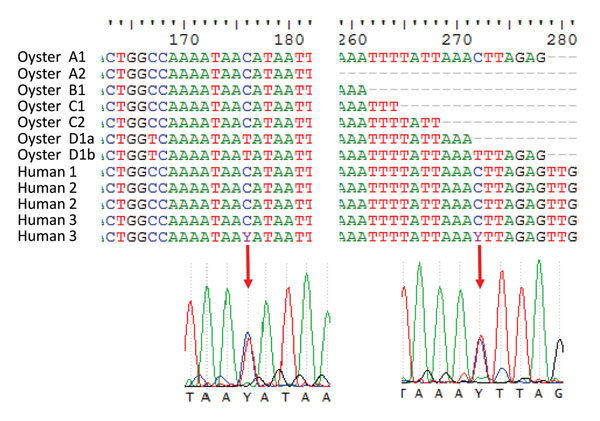
Alignment of capsid genes of noroviruses isolated from humans and oysters in Denmark, showing regions in which sequence differences were detected. The chromatograms show the mixed bases in human 3 sample 2. Two reverse transcription PCR products of different size were identified in oyster sample D1; however, no apparent sequence differences were identified in the 2 products (D1a and D1b). Human 1 submitted only 1 fecal sample.

Since the emerging of GII.P17-GII.17 in Asia, sporadic cases have been reported worldwide (*3**,**9*). In this study, we established a direct molecular link between a common food source and a series of acute gastroenteritis outbreaks. Even though these represent European outbreaks, our results show that oysters act as vehicles for the rapid spread of emerging noroviruses to distant geographic areas. Furthermore, we document that quasispecies of GII.P17-GII.17 might occur simultaneously in a host. This finding should be considered in future molecular-epidemiologic outbreak investigations.
